# Advancing Food Security with Farmed Edible Insects: Economic, Social, and Environmental Aspects

**DOI:** 10.3390/insects16010067

**Published:** 2025-01-11

**Authors:** José E. Aguilar-Toalá, Abraham M. Vidal-Limón, Andrea M. Liceaga

**Affiliations:** 1 Departamento de Ciencias de la Alimentación, División de Ciencias Biológicas y de la Salud, Universidad Autónoma Metropolitana, Unidad Lerma, Av. de las Garzas 10, Col. El Panteón, Lerma de Villada 52005, Estado de México, Mexico; j.aguilar@correo.ler.uam.mx; 2 Red de Estudios Moleculares Avanzados, Clúster Científico y Tecnológico BioMimic^®^, Instituto de Ecología A.C. (INECOL), Carretera Antigua a Coatepec 351, El Haya, Xalapa 91073, Veracruz, Mexico; abraham.vidal@inecol.mx; 3 Protein Chemistry and Bioactive Peptides Laboratory, Purdue University, 745 Agriculture Mall, West Lafayette, IN 47907, USA

**Keywords:** farmed edible insects, insect biomass production, entomophagy, food security, sustainability

## Abstract

Farmed edible insects are considered a potential resource to help address food security concerns toward the year 2050. The sustainability (e.g., lower environmental impact), nutritional (e.g., high-quality proteins, essential amino acids, fiber, unsaturated fats, vitamins, and minerals) and health (e.g., antioxidant, antihypertensive, anti-inflammatory, antimicrobial, and immunomodulatory) benefits are the main reasons for the rise in interest for insects as alternative protein sources for food and feed production. Thus, edible insects can address the future global protein demand of an ever-increasing world population. In this context, several aspects related to their sustainability have been explored and addressed from an environmental perspective. This review describes the rationale for using insects as alternative protein sources and provides a comprehensive viewpoint, integrating economic, environmental, and social aspects into their sustainability framework toward addressing food insecurity concerns. For example, edible insects offer a more sustainable protein source comparable to, or even better than, that of conventional livestock. Considering their sustainability advantages, insects are noted for their lower impact on natural resources (e.g., water and agrarian land) and lower greenhouse gas emissions (e.g., carbon dioxide and methane). From a socioeconomic point of view, edible insects have lower production costs compared to conventional animal protein sources because of their high feed efficiency conversion, rapid growth rate, and short life cycles. Currently, the market for edible, farmed insects is becoming a significant economic activity that not only meets the needs of industry and consumers but also supports the ability of future generations to maintain a secure and sustainable community.

## Simple Summary

Edible insects are a promising alternative food source to address future food security issues. In addition to their high nutritional composition and health benefits, reared insects have more sustainable production processes compared to traditional livestock farming. The growing market for farmed insects supports both current industry needs and future food security challenges. Overall, this review discusses the sustainability of edible insects as alternative protein sources, integrating economic, environmental, and social aspects.

## 1. Introduction

Insect consumption (i.e., entomophagy) has emerged as a potential solution to future food problems worldwide, such as malnutrition, food shortage problems, and increased animal protein demand [1,2]. In this context, it was projected that by the year 2050, the world food demand will increase by 50%, with a simultaneous increase in the demand for animal-based protein by 70% [1]. The global edible insect market is showing interest in this novel protein source, as evidenced by several startup companies and a number of scientific publications in the last decade, with market trends leading toward a global edible insect market size of approximately USD 8 billion in the next 10 years [3]. Edible insects have also drawn attention in the food and feed industries because of their associated nutritional and health benefits [4,5]. Overall, it has been well-documented that edible insects can provide nutrients of high quality, such as protein, essential amino acids, fiber, mono- and polyunsaturated fats, vitamins, and minerals, even at levels comparable to those of animal protein sources from conventional livestock species [2,6]. In addition, edible insects can offer some health benefits, including antioxidant, antihypertensive, anti-inflammatory, antimicrobial, and immunomodulatory activities, which are associated with their content of bioactive compounds, such as phenolic compounds, chitin, chitosan, fatty acids, and bioactive peptides [7,8,9], that result in nutritional and health benefits to consumers [4,10].

In addition to their nutritional and health benefits, insects have been proposed as a sustainable food system alternative to conventional livestock species, providing food for humans and feed for animals with lower sustainability impacts. For example, in farmed stock animals (e.g., poultry and pigs), insect-based feed can act as substitutes of soybean meal, which has been related to some metabolic issues in these animals [11]. Based on the above information, this review provides an overview of the economic, social, and environmental aspects of farmed edible insects to further utilize this sustainable resource and contribute to more sustainable diets and/or food and feed alternatives. 

## 2. Sustainability Definition and Scope in Food Production

**Figure 1:**
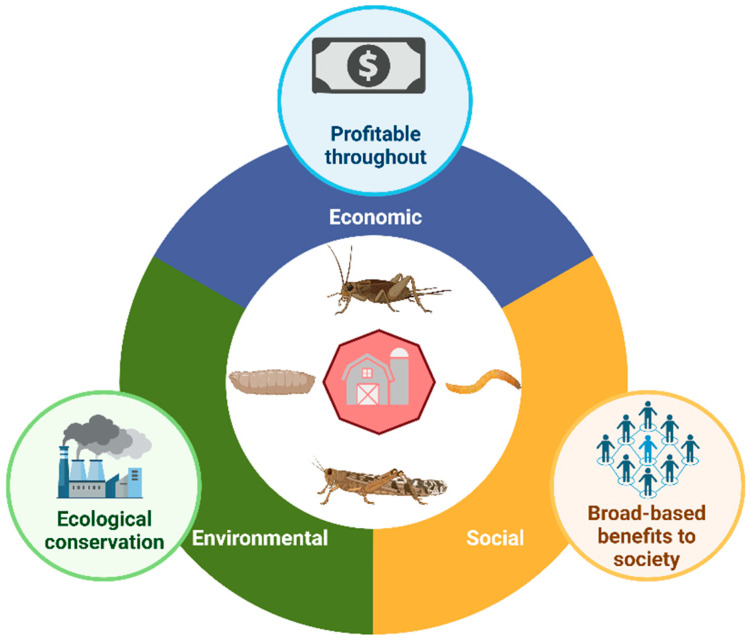
Dimensions of sustainability aspects of farmed edible insects related to an economic, environmental and social approach. Figure created with BioRender.com.

Although most scientific literature addresses the topic of sustainability from an environmental (ecological) point of view, it is also important to address it from an integral approach that includes social and economic aspects (Figure 1). All three aspects (environmental, social, and economic) comprise the multiple dimensions of sustainability since consumers have become more concerned with environmental issues since the 1980s, particularly regarding global climate change issues [12]. The Food and Agriculture Organization (FAO) and US Department of Agriculture (USDA) have mentioned that a major critical challenge toward ensuring food security is related to food production without causing a significant increase in greenhouse gas emissions [13,14]. However, it is important to note that the COVID-19 pandemic had a positive environmental effect due to the imposed restrictions and significant slowdown of socioeconomic activities, resulting in better air quality in many cities that was accompanied by a reduction in water pollution. However, it is unclear whether this positive trend is continuing or if will have any long-term impact [15]. As mentioned above, according to the FAO [16], a sustainable food system must involve economic, social, and environmental sustainability aspects. This approach indicates that a sustainable food system should be profitable throughout (economic) whilst having broad benefits to society (social), as well as having a positive or neutral impact on the planet (environmental). In addition, the overlays of two of the above aspects generate new interactions. For example, the overlay of social and economic aspects generates an inclusive growth, while social and environmental aspects generates eco-social progress, and economic and environmental aspects creates what is referred to as a green growth [17]. Studies indicate that a sustainable agri-food system should be economically viable and efficient; socially responsible regarding farmers, workers, communities, consumers, and society; and also ecologically sustainable [18]. In this context, the application of insect biomass production as a sustainable food and feed system should guarantee food security and nutrition for all of society [19].

## 3. Economic and Social Aspects of Farmed Edible Insects

The potential socio-economic impact of farmed edible insects on improving food security, primarily in low- and middle-income countries, is highlighted by the fact that entomophagy is already practiced in many of these countries. The main economic factor of farmed edible insects is that they have the potential to become a commodity in global markets, thus leading to a complex industry with many job opportunities for people, providing means of livelihood to families, and making insect farming a promising strategy for alleviating poverty [19,20,21]. It is expected that the edible insect market will grow, which can improve the livelihoods of farmers and people related to this economic activity [20,22]. 

Furthermore, insect biomass production can supply the current shortages of conventional food sources because insects have more availability, particularly in rural areas, and low prices; at the same time, they can address some of the dietary needs of populations [23]. In this context, concerning social matters, farmed edible insects can mitigate malnutrition problems globally by providing food with the highest quality (e.g., protein, essential amino acids, fiber, mono- and polyunsaturated fats, vitamins, and minerals) with broad availability and access [24]. Particularly, in some countries, edible insects have an important economic value, which, for many communities and ethnic groups, represents an important part of their income [24]. For example, insect farming in East Africa is considered an emerging business for developing “climate-smart protein” and profitable income. It is reported that over 80% of feed processors in East Africa are willing to use insects as animal feed for their livestock and fish farms [25]. 

Other economic aspects on the application of edible insects include their overall production cost, which encompasses the valorization of various agricultural side-streams and by-products from the agro-industrial sector by using them as potentially effective raw materials for bioconversion by edible insects [26]. For example, Vinci et al. [27] reported that the cost associated with commercial mealworm production was USD 7.53 per kg of mealworm protein, whereas that associated with edible pork protein was USD 59.04 per kg, making it approximately eight times more expensive to produce pork than edible insects. In this sense, the feed used for farmed insects can be from resources that comprise materials from agricultural side-streams, which can be used as low-cost raw materials; hence, their expense of production can be decreased. 

Regarding these rearing substrate resources, it is also possible to use leftover food generated from different agricultural production stages, postharvest handling and storage, food industry/commercial food production, wholesale and retail trade distribution, large-scale kitchens, and even household food biowaste [28,29]. The above statement agrees with a specific strategy to tackle the food waste problem; the core of this strategy is based on the three R´s of waste management: reduce, reuse, and recycle. In fact, the use of food waste or food processing by-products as feed for edible insects has been considered as a form of insect bioconversion to produce not only human food and animal feed but also other outputs such as fertilizers and secondary industrial compounds (e.g., biofuel, emollients, pharmaceuticals, and dyes) [28,30]. In contrast, it is more economical to produce farmed insects than most conventional livestock. For example, Vinci, Prencipe, Masiello, and Zaki [27] found that the cost to produce 1 kg of mealworm protein, in terms of the depletion of fossil resources, was USD 6.63 per kg, while the cost to produce the same amount (1 kg) of pork protein was of USD 47.28 per kg. This highlights the potential for farming edible insects as a profitable economic activity. With these arguments, the production system of farmed insects can be considered within a circular economy, providing an attractive key for closing the loop of their food value chain [28]. Thus, it is expected that considerable growth in this sector will bring jobs, novel commodities, and new inputs to the food and feed chain; the simultaneous reduction and reuse of wastes is currently considered a global problem [30]. Nevertheless, we must ensure that insect rearing does utilize rearing substrates (e.g., soybean meal, poultry feed, and aqua feed) that are already in demand as animal feed in conventional livestock, broiling, and fish farming. Therefore, by-products and other low-cost rearing substrates need to be further considered.

Another important aspect related to the profitability of insect biomass production relates to the economic figures associated with the four major insect species currently farmed for human food markets, particularly in the European Union, where they are considered novel foods. These four species include the lesser mealworm (Alphitobius diaperinus), yellow mealworm (Tenebrio molitor), house cricket (Acheta domesticus), and migratory locust (Locusta migratoria). In addition, the black soldier fly (Hermetia illucens) is approved for use in animal feed production. According to Niyonsaba et al. [31], the price for dried Hermetia illucens larvae can range from EUR 1.82 to EUR 18.90 per ton of product; whereas the price for dried Alphitobius diaperinus larvae is listed at EUR 118.00 per ton of product. Authors attribute this higher sales price to costly technologies, such as freeze-drying (lyophilization), which is used to process the larvae. In contrast, the sale price for dried Tenebrio molitor larvae ranges from EUR 5.28 to EUR 97.00 per ton of product, with the higher prices given to Tenebrio molitor samples aimed for human consumption. Finally, for Acheta domesticus, the economic figures show sale prices between EUR 45.46 and EUR 200.00 per ton of dried product, with the higher price being derived from crickets reared exclusively for human consumption [31]. It is worth mentioning that the sale prices listed above are only estimations based on available literature, and these are dependent of the market’s geographic location and the end-product target market sector (i.e., animal feed or human food). 

In 2022, it was estimated that, in the United States and Canada, there were 21 and 41 insect biomass producers, respectively, while there were 22 companies offering insect-based products in Canada and only one in the United States [32]. In Europe, as of 2023, there were about 244 insect farming tech startups. Based on the 500 tons of insect-based products (whole insects, insect ingredients, etc.) in 2019, it is predicted that the European market will expand to 260,000 tons by 2030 [33]. Furthermore, the worldwide market of insect farming is expected to grow by 4.3% (Compound Annual Growth Rate) from USD 1.5 billion in 2023 to around USD 35.3 billion by 2033 [34]. Particularly, this expansion trajectory is fueled by increasing demand from industries utilizing insects for obtaining protein, animal feed supplements, and sustainable food alternatives. The market value of edible insects worldwide (2023) was dominated by the Asia-Pacific (40.4%) region, followed by the Europe (22.1%), Latin America (21.2%), and North America (13.0%) regions, and, to a lesser extension, by the Middle East and Africa [35].

### Insect-Based Products in the Market

In recent years, a diverse range of insect-based products has emerged in the market for foods that meet consumer preferences and nutritional needs. Traditionally, insects were consumed as whole entities, either dried or roasted; however, there has been a significant evolution in their culinary applications. Today, insect-based protein powders are gaining traction as versatile ingredients in energy bars, snacks, pasta, and baked goods, enhancing their nutritional profile and offering unique flavours [36]. Table 1 highlights how insect-based products have become increasingly specialized to serve multiple sectors (e.g., human food, animal feed, and agriculture), emphasizing the adaptability of insects as a sustainable protein source. North American companies appear to focus on crickets and black soldier fly larvae for consumer-friendly food products, leveraging the nutritional benefits of crickets as high-protein, low-fat options. In contrast, European companies like to show a more integrated approach, offering not only food but also advanced animal feed and plant fertilizers derived from insects, reflecting Europe’s broader acceptance of sustainable insect-based innovations. For instance, some companies specialize in cricket protein powder (Table 1), providing a sustainable protein source for health-conscious consumers. The most recent category within insect-based products are insect-derived oil and fats, containing omega-6 fatty acids and variable amounts of omega-3 fatty acids, depending on the insect order, species, life cycle stage, and diet, among others [37,38,39,40]. Mealworms and black soldier fly larvae are at the forefront of producing these oils, showcasing the potential of insects as a source of healthy fats [39].

Beyond human consumption and animal feed, the exploration of insect-based products for agricultural use has gained momentum. Insect frass, a by-product of insect farming, is increasingly being recognized for its potential as a natural fertilizer. This product not only nourishes plants with essential nutrients but also promotes microbial growth in the soil, enhancing soil structure through organic matter [41]. Some companies have developed insect-based fertilizers, capitalizing on the benefits of black soldier fly larvae frass and highlighting the multifunctionality of insect biomass production. As consumers become more environmentally conscious and seek sustainable alternatives, the diverse range of insect-based products available, ranging from protein-rich powders to eco-friendly fertilizers, demonstrates the potential of insects to contribute significantly to both food security and sustainable agricultural practices.

## 4. Environmental Sustainability

**Figure 2:**
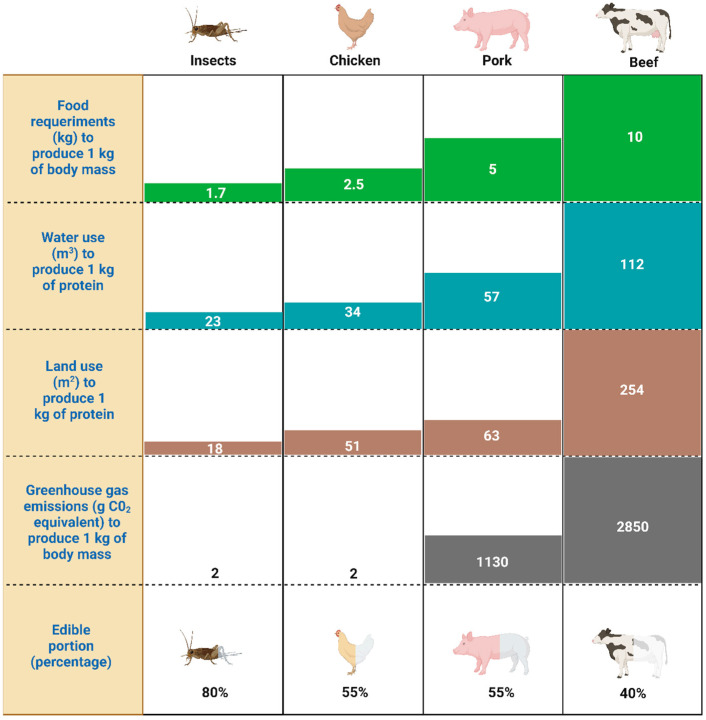
Comparison chart of resources used in insect biomass and livestock species production. Data adapted from Liceaga [44], Ros-Baró, Casas-Agustench, Díaz-Rizzolo, Batlle-Bayer, Adrià-Acosta, Aguilar-Martínez, Medina, Pujolà and Bach-Faig [45], Dobermann, Swift and Field [54], and Ooninc et al. [66]. Figure created with BioRender.com.

As outlined above, environmental/ecology sustainability is the most studied aspect related to the sustainability of farmed insects. This is because insects consume fewer resources per output of animal protein produced or body mass gained at a significant level [42]. In this context, there are key physiological and biological differences between conventional livestock species and insects that positively contribute to the lower environmental impact derived from insect production. Edible insects are physiologically and biologically different from livestock species, mainly by their metabolism. Moreover, the extent of the environmental impact of conventional livestock varies depending on the type of species, farming system/production method, level of feed consumption, production period, as well as by the composition and nutritional value of the feed provided [43]. It is well-documented that farmed edible insects require fewer resources to produce the same quantity of protein or body mass compared with conventional livestock species production processes, such as chicken, pork, and beef (Figure 2). Insect farming uses much less land and water requirements than those used for farming conventional livestock, which means that reared insects can thrive in confined spaces, be reared indoors in vertical farms, and under drought conditions. For example, farmed insects use about 1.5-, 2.5-, and 5-fold less water than poultry, pork, and beef, respectively, to produce 1 kg of protein [44,45]. In fact, some insects (i.e., *Tenebrio molitor*) are reported to not require additional drinking water when reared under appropriate conditions of humidity and when provided with a specific diet (e.g., carrots and bran/grain) [46]. Similarly, some insects that live in the desert (e.g., beetles) can survive on metabolic water only (i.e., water content in their feed) [47]. Additionally, water is not only used for the direct consumption of livestock species but is also used to clean housing pens/units, wash animals, and cool down facilities and animals, which, in the case of farmed edible insects, may be lower or even null in some of these activities [48].

Similarly, regarding agricultural land use, poultry and pork use nearly 3.5 times more land than insects, while for beef, this value is ca. 14-fold greater [44,45]. Moreover, it is especially necessary to take into account the land required for producing feed, either by grazing planted pasture or by planting crops depending on the conventional livestock species [48]. The above causes deforestation and biodiversity losses from the expansion of pasture and feed crops [43]. Accordingly, edible insects not only require less land to live and eat but also use less land for producing their feed substrate. Thus, insect production is also efficient in terms of land use compared with conventional livestock species. These resources are very important because the degradation of land and water qualities due to human-related activities has become a pressing global issue, putting our natural resources at risk around the world [49]. In this sense, the implementation of farmed edible insects is proposed as an alternative option in order to prevent global issues related to the preservation of land and water [50]. 

Several researchers [51,52,53] have indicated that another important parameter, the feed conversion efficiency (FCE), must also be considered when selecting environmentally sound food alternatives. It is reported that insects have a better feed conversion ratio compared with conventional livestock. For example, insects have a feed conversion ratio of 1.7 that is at least equal to or threefold as efficient compared to poultry and pork (1.7 and 5.0, respectively), and six times more efficient than beef [45,54]. It is important to note that the FCE varies significantly among insect species. For instance, van Huis and Oonincx [55] reported a higher FCE in mealworms (45%) and black soldier fly (55%), who convert dietary protein to edible body mass. Similarly, Ramos-Elorduy et al. [56] found that *Tenebrio molitor* shows 53–73% of the FCE compared to livestock species (10–12%). Thus, edible insects are considered more efficient at converting feed into animal protein and feed energy into food energy. The above statement is explained by the fact that insects are poikilothermic (cold-blooded), so their metabolism (energy) is not used to maintain their body temperature. This also means that they do not rely on water evaporation as a mechanism for temperature regulation, leading to a higher feed conversion ratio [23]. However, concerns can arise with the effect of energy requirements needed to maintain optimal rearing temperatures in facilities used for insect biomass production, particularly in cold climate countries. One solution being proposed is to utilize the excess heat generated from Information and Communication Technology (ICT) data centers, where the electricity used to run equipment is transformed into heat, then cooled off and exhausted to the environment in a temperature range of 30–45 °C [57]. These authors demonstrated that the excess heat (at 30 °C and relative humidity of 50%) from such centers can be successfully utilized as a heating source for mealworm biomass production, from egg to fully developed larvae in less than 56 days [57].

In addition, the FCE can explain the relatively low requirements in terms of land and water used in insect production chains compared to respective livestock species [55]. As previously mentioned, another advantage in relation is that edible insects can feed on waste or by-products from agricultural practices and the food industry [53]. The diet of insects commonly includes plant resources (e.g., wheat bran, cereal, oats, soy, corn, chicken mash), fruits and vegetables wastes (e.g., carrots, cabbage, pineapple, banana), and microbial resources (e.g., beer yeast spent brewers) [26,58], which in turn can affect the final nutrient composition of edible insects, in particular, their protein and fat contents and their profiles [58]). For example, it has been observed that protein-rich substrates enhance the system of the insect’s FCE [59]. Some authors [52,60] have reported that mealworms fed a diet composed with a mix of dried by-products from beer brewing, bread/cookie production, and potato processing by-products develop well and contain a fairly constant nutrient composition. It is important to note that although insects have the potential to convert organic by-products into food/feed, the limited evidence related to the safety of the final products has resulted in EU regulations still prohibiting the use of waste (by-) products as a substrate for growing edible insects [61]. Therefore, when selecting the choice of organic waste management for insect biomass production, considerations about their sourcing consistency (i.e., certain crops available seasonally) and its effects on production yield and safety need to be carefully evaluated.

Other notorious advantages of insect biomass production include an overall faster growth rate, short development cycle, high survival of juvenile stages, high level of egg-laying capacity, and high daily growth potential of biomass [62]. Despite the aforementioned advantages, it is important to note that cricket farms experience higher mortality rates compared to conventional livestock farms, primarily due to factors such as high population density and the cannibalistic behaviors observed in crickets. Unlike mammals, where mortality is often managed through veterinary care and controlled environments, cricket farming faces unique biological challenges [63,64]. Another potential issue is related to the presence of viruses in mass-reared edible insects that could have different consequences, ranging from asymptomatic infection to entire colony collapse [65]. Figure 2 summarizes the competitive advantages of edible insects compared with conventional livestock species, such as poultry, pork, and beef. Additionally, it is important to note that in the context of insects serving as food, we consider a majority of their body to be edible (80–100%), while in the case of conventional livestock species, only about 40–55% of the animal is consumed as food, suggesting that more edible mass is available from insects, which can lead to a significant reduction in food waste and loss. 

Generally, the most recognized environmental assessment method for evaluating sustainability in foods is the life cycle assessment (LCA) method [67]. This method quantitatively determines the inputs, energy flows, and environmental impact of systems, products, and services through the entire chain [55]. This tool has been applied to farmed edible insects in recent years, primarily over the last decade. A summary of recent studies that applied LCA to farmed insects is shown in Table 2.

The life cycle assessment studies listed in Table 2 report the environmental impacts of insect production at different scales for use as food and feed. In the majority of cases, life cycle assessments have been applied toward black soldier fly (*Hermetia illucens*) and yellow mealworm (*Tenebrio molitor*) production systems and, to a lesser extent, cricket (*Gryllus bimaculatus* and *Acheta domesticus*) production systems. For example, Halloran et al. [68] and Dreyer et al. [69] compared the environmental impacts of cricket (*Gryllus bimaculatus* and *Acheta domesticus*) and mealworm (*Tenebrio molitor*) production systems, respectively, to that of poultry. The authors reported that protein obtained from these two insects has a lower environmental impact than that obtained from poultry farming. Similarly, Smetana et al. [70] found that black soldier fly (*Hermetia illucens*) production is twice as environmentally efficient as poultry meat. Another study reported that a black soldier fly (*Hermetia illucens*) production system shows higher energy conversion efficiency, where feed is more efficiently converted into insect weight gain [59]. Additionally, Joensuu and Silvenius [71] and Vinci, Prencipe, Masiello, and Zaki [27] reported that the global warming potential from farmed insects is low compared to traditional livestock production in black soldier fly (*Hermetia illucens*) and mealworm (*Tenebrio molitor*) systems. These studies highlight how insect biomass production can have lower impacts on diverse environmental parameters, especially when compared to other food and feed production systems.

**Table 2:** Life cycle assessment studies about the environmental impact of farmed edible insects as food and feed.

Edible Insect System [Geographical Region]	System Boundaries	Functional Unit *	Principal Findings	References
Mealworm (*Tenebrio molitor*) in farm-scale facilities [Italy]	Cradle-to-slaughterhouse gate	Nutritionally based (kg of protein on dry matter basis)	Overall, insects have lower environmental impacts, particularly on land use, climate-altering emissions (global warning and ozone formation), and fossil resources than pork production	[27]
Mealworm (*Tenebrio molitor*) in small-scale organic production facilities [Austria]	Cradle-to-gate	Nutritionally based (kg of protein on dry matter basis)	Overall, insects have lower environmental impacts (18–72% less impacts) than organic broiler production	[69]
*Protaetia brevitarsis seulensis* in small-scale facilities [South Korea]	Cradle-to-gate	Mass based (kg of dried insect, protein, and fat)	Overall, insects have lower environmental impacts, particularly on land use, mineral extraction, aquatic and terrestrial ecotoxicity	[72]
Black soldier fly (*Hermetia illucens*) in highly productive pilot plants [Netherlands/Switzerland]	Cradle-to-gate	Mass based (kg of dried and pelletized organic fertilizer, fresh biomass [puree], protein concentrated meal, and fat)	Insect production has the potential to be a more sustainable protein, fertilizer, and lipid production source Fresh insect biomass is almost twice as sustainable as fresh chicken meat	[70]
Field (*Gryllus bimaculatus*) and house (*Acheta domesticus*) crickets in a medium-scale production system [Thailand]	Cradle-to-farm gate	Mass based (kg of edible mass) and nutritionally based (kg of protein in edible mass)	Overall, both crickets’ production systems have lower environmental impacts when compared to industrial broiler farming systems	[68]
Black soldier fly (*Hermetia illucens*) in a small-scaled production system [West Africa]	Cradle-to-gate	Mass based (kg of larvae meal on dry matter basis)	The insect system shows the highest conversion efficiency	[59]
Black soldier fly (*Hermetia illucens*) in a pilot plant [Italy]	Cradle-to-gate	Mass based (ton of food waste, kg of protein and kg of lipids)	The insect bioconversion system has less land use compared to soymeal and rapeseed oil systems	[73]
Black soldier fly (*Hermetia illucens*) in an industrial-scale facility [Netherlands/Finland]	Cradle-to-gate	Mass based (kg fresh mealworms)	The global warming potential (CO_2_ equivalents emissions) is low compared to traditional livestock production	[71]

* The functional unit is often based on the mass or component of the product under study.

## 5. Conclusions

Edible insects have been consumed since ancient times and continue to provide food for millions of people globally. In recent years, insects have been recognized by their nutritional (e.g., high-quality proteins, essential amino acids, fiber, unsaturated fats, vitamins, and minerals) and health-related (e.g., antioxidant, antihypertensive, anti-inflammatory, antimicrobial, and immunomodulatory) properties. However, in addition to these benefits, farmed insects have demonstrated economic, environmental, and social benefits that promote them as sustainable food systems that can advance food security. Several activities associated with their production generate jobs and income, providing a means of livelihood to families and ensuring zero hunger, especially in low- and middle-income countries. This is confirmed by the fact the edible insect market is in constant growth. Nevertheless, careful consideration needs to be taken toward current rearing substrates (e.g., organic waste material) being used for insect biomass production in order to avoid limiting feed substrate accessibility for livestock and other farmed animals, such as broilers and fish. In addition, regulatory guidelines on the use of rearing substrates differ by country or region, which can influence global insect biomass production, as well as affect the safety of the final food/feedstuffs.

The future of insect biomass production systems will rely on optimizing current production systems, promoting and facilitating rearing practices to increase socio-economic outputs, incentivize farmers toward the adoption of insect biomass production, and finally, promote edible insects as food and feed based on their benefits to the environment and society.

## Figures and Tables

**Table 1:** Overview of global companies offering insect-based products as of 2023.

Company	Insect-Based Products Offers			Webpage ^1^	Country
	Food	Feed	Plants		
Entomo Farms	Cricket (powder and whole roasted)			https://entomofarms.com/	Canada
Protix B.V.		Insect-based protein meal, insect oil, insect puree, and products derived from black soldier fly larvae	Insect-based fertilizer from soldier fly larvae frass	https://protix.eu/	The Netherlands
Fair Insects BV (acquired by Protix in 2017)	Mealworm, crickets, and locusts				The Netherlands
Ÿnsect	Fiber textured insect protein, hydrolysed protein powder, and oil derived from mealworm	Mealworm larvae powder and protein hydrolysate	Insect-based fertilizer from mealworm frass	https://www.ynsect.com/	France
CRIK Nutrition	Cricket protein powder			https://buyhi.co/	Canada
Beta Hatch		Mealworm (meal, oil, and whole dried)	Insect-based fertilizer from mealworm frass	https://betahatch.com/	United States
Exo	Cricket (powder, protein and breakfast bars, and cookies and bites)			https://exoprotein.com/	United States
Grubby Farms		Whole dried black soldier fly larvae			United States
Wilder Harrier		Processed black soldier fly larvae			Canada
Agronutris- SAS EAP Group	Whole dried mealworm larvae, powder, and protein products, biscuits, and pasta-based products	Processed black soldier fly larvae	Insect-based fertilizer from soldier fly larvae frass	https://www.agronutris.com/en/	France
Cricket One Co., Ltd.	Whole dried crickets, protein powders, texturized protein			https://www.cricketone.asia/	Vietnam
Nutriearth	Vitamin D3 from mealworm			https://www.nutriearth.fr/	France
Reploid Group AG		Black soldier fly larvae			Austria
Entogama	Whole dried crickets and mealworms; cricket and mealworm powders			https://www.entogama.com/	Lithuania
Global Bugs Asia	Cricket powder	Cricket powder		https://globalbugs.asia/	Thailand
Aspire Food Group	Cricket powder	Cricket powder	Cricket frass	https://aspirefg.com/	USA, Canada
Nutrinsect	Lyophilized cricket powder	Whole dried crickets, cricket powder, and pellets		https://en.nutrinsect.it/	Italy
Armstrong Cricket Farm		Crickets, mealworms, superworms, and hornworms		https://armstrongcrickets.com/	USA

^1^ websites last accessed on 12 December 2024.

## Data Availability

No new data were created or analyzed in this study.

## References

[1] Searchinger T., Waite R., Hanson C., Ranganathan J., Dumas P. (2019). Creating a Sustainable Food Future—A Menu of Solutions to Feed Nearly 10 Billion People by 2050.

[2] Kim T.K., Yong H.I., Kim Y.B., Kim H.W., Choi Y.S. (2019). Edible insects as a protein source: A review of public perception, processing technology, and research trends. Food Sci. Anim. Resour..

[3] Liceaga A.M., Aguilar-Toalá J.E., Vallejo-Cordoba B., González-Córdova A.F., Hernández-Mendoza A. (2022). Insects as an alternative protein source. Annu. Rev. Food Sci. Technol..

[4] Aguilar-Toalá J.E., Cruz-Monterrosa R.G., Liceaga A.M. (2022). Beyond human nutrition of edible insects: Health benefits and safety aspects. Insects.

[5] da Silva Lucas J.A., Menegon de Oliveira L., da Rocha M., Prentice C. (2020). Edible insects: An alternative of nutritional, functional and bioactive compounds. Food Chem..

[6] Orkusz A. (2021). Edible insects versus meat—Nutritional comparison: Knowledge of their composition is the key to good health. Nutrients.

[7] Hall F., Reddivari L., Liceaga A.M. (2020). Identification and characterization of edible cricket peptides on hypertensive and glycemic in vitro inhibition and their anti-inflammatory activity on RAW 264.7 macrophage cells. Nutrients.

[8] Malm M., Liceaga A.M. (2021). Physicochemical properties of chitosan from two commonly reared edible cricket species, and its application as a hypolipidemic and antimicrobial agent. Polysaccharides.

[9] Nino M.C., Reddivari L., Osorio C., Kaplan I., Liceaga A.M. (2021). Insects as a source of phenolic compounds and potential health benefits. J. Insects Food Feed..

[10] Nowakowski A.C., Miller A.C., Miller M.E., Xiao H., Wu X. (2022). Potential health benefits of edible insects. Crit. Rev. Food Sci. Nutr..

[11] Beller S., Grundmann S.M., Pies K., Most E., Schuchardt S., Seel W., Simon M.-C., Eder K., Ringseis R. (2024). Effect of replacing soybean meal with *Hermetia illucens* meal on cecal microbiota, liver transcriptome, and plasma metabolome of broilers. Poult. Sci..

[12] Choi S., Ng A. (2011). Environmental and economic dimensions of sustainability and price effects on consumer responses. J. Bus. Ethics.

[13] Ingram J., Ericksen P., Liverman D. (2012). Food Security and Global Environmental Change.

[14] FAO (2006). Livestock Report 2006.

[15] Rume T., Islam S.M.D.-U. (2020). Environmental effects of COVID-19 pandemic and potential strategies of sustainability. Heliyon.

[16] FAO (2018). Sustainable Food Systems: Concept and Framework.

[17] FAO (2014). Developing Sustainable Food Value Chains. Guiding Principles.

[18] Bachev H., Ivanov B., Toteva D., Sokolova E. (2017). Agrarian sustainability in Bulgaria—Economic, socual and ecological aspects. Bulg. J. Agric. Sci..

[19] Lange K.W., Nakamura Y. (2023). Potential contribution of edible insects to sustainable consumption and production. Front. Sustain..

[20] Guiné R.P.F., Correia P., Coelho C., Costa C.A. (2021). The role of edible insects to mitigate challenges for sustainability. Open Agric..

[21] Aidoo O.F., Osei-Owusu J., Asante K., Dofuor A.K., Boateng B.O., Debrah S.K., Ninsin K.D., Siddiqui S.A., Chia S.Y. (2023). Insects as food and medicine: A sustainable solution for global health and environmental challenges. Front. Nutr..

[22] Guiné R.P.F., Florença S.G., Anjos O., Correia P.M.R., Ferreira B.M., Costa C.A. (2021). An insight into the level of information about sustainability of edible insects in a traditionally non-insect-eating country: Exploratory study. Sustainability.

[23] Matandirotya N.R., Filho W.L., Mahed G., Maseko B., Murandu C.V. (2022). Edible insects consumption in Africa towards environmental health and sustainable food systems: A bibliometric study. Int. J. Environ. Res. Public Health.

[24] Cerritos R. (2009). Insects as food: An ecological, social and economical approach. CABI Int..

[25] Tanga C.M., Egonyu J.P., Beesigamukama D., Niassy S., Emily K., Magara H.J., Omuse E.R., Subramanian S., Ekesi S. (2021). Edible insect farming as an emerging and profitable enterprise in East Africa. Curr. Opin. Insect Sci..

[26] El Hajj R., Mhemdi H., Besombes C., Allaf K., Lefrançois V., Vorobiev E. (2022). Edible insects’ transformation for feed and food uses: An overview of current insights and future developments in the field. Processes.

[27] Vinci G., Prencipe S.A., Masiello L., Zaki M.G. (2022). The application of life cycle assessment to evaluate the environmental impacts of edible insects as a protein source. Earth.

[28] Ojha S., Bußler S., Schlüter O.K. (2020). Food waste valorisation and circular economy concepts in insect production and processing. Waste Manag..

[29] Edjabou M.E., Petersen C., Scheutz C., Astrup T.F. (2016). Food waste from Danish households: Generation and composition. Waste Manag..

[30] Fowles T.M., Nansen C., Närvänen E., Mesiranta N., Mattila M., Heikkinen A. (2020). Insect-based bioconversion: Value from food waste. Food Waste Management: Solving the Wicked Problem.

[31] Niyonsaba H., Höhler J., Kooistra J., Van der Fels-Klerx H., Meuwissen M. (2021). Profitability of insect farms. J. Insects Food Feed..

[32] Larouche J., Campbell B., Hénault-Éthier L., Banks I.J., Tomberlin J.K., Preyer C., Deschamps M.H., Vandenberg G.W. (2023). The edible insect sector in Canada and the United States. Anim. Front..

[33] Skotnicka M., Karwowska K., Kłobukowski F., Borkowska A., Pieszko M. (2021). Possibilities of the development of edible insect-based foods in Europe. Foods.

[34] MARKET.US Global Insect Farming Market by Insect Type (Crickets, Mealworms, Black Soldier Flies, House Flies, Others), by Application (Animal Feed, Fertiliser, Protein, Biofuels, Others), by End-Use (Food and Feed, Agricultural, Pharmaceuticals, Biotechnology, Others), by Region and Companies—Industry Segment Outlook, Market Assessment, Competition Scenario, Trends and Forecast 2024–2033. https://market.us/report/insect-farming-market/#overview.

[35] STATISTA Market Value of Edible Insects Worldwide in 2018 and 2023, by Region (in Million U.S. Dollars). https://www.statista.com/statistics/882360/edible-insects-market-size-global-by-region/.

[36] Gori A., Armani A., Pedonese F., Benini O., Mancini S., Nuvoloni R. (2025). Heavy metals (Pb, Cd, Ni) in insect-based products for human consumption sold by e-commerce in the EU market: Occurrence and potential health risk associated with dietary exposure. Food Control.

[37] Sosa D.A.T., Fogliano V. (2017). Potential of insect-derived ingredients for food applications. Insect Physiol. Ecol..

[38] Yap J.W.-L., Lee Y.-Y., Tang T.-K., Chong L.-C., Kuan C.-H., Lai O.-M., Phuah E.-T. (2023). Fatty acid profile, minor bioactive constituents and physicochemical properties of insect-based oils: A comprehensive review. Crit. Rev. Food Sci. Nutr..

[39] Riekkinen K., Väkeväinen K., Korhonen J. (2022). The effect of substrate on the nutrient content and fatty acid composition of edible insects. Insects.

[40] Rumpold B.A., Schlüter O.K. (2013). Nutritional composition and safety aspects of edible insects. Mol. Nutr. Food Res..

[41] PROTIX (2024). All-Natural Sustainable Insect Fertiliser—Flytilizer for Plant Care. https://protix.com/products/flytilizer-for-plant-care.

[42] Berggren A., Jansson A., Low M. (2019). Approaching ecological sustainability in the emerging insects-as-food industry. Trends Ecol. Evol..

[43] Halloran A., Hansen H.H., Jensen L.S., Bruun S., Halloran A., Flore R., Vantomme P., Roos N. (2018). Comparing environmental impacts from insects for feed and food as an alternative to animal production. Edible Insects in Sustainable Food Systems.

[44] Liceaga A.M., Wu J. (2022). Chapter Four-Edible insects, a valuable protein source from ancient to modern times. Advances in Food and Nutrition Research.

[45] Ros-Baró M., Casas-Agustench P., Díaz-Rizzolo D.A., Batlle-Bayer L., Adrià-Acosta F., Aguilar-Martínez A., Medina F.-X., Pujolà M., Bach-Faig A. (2022). Edible insect consumption for human and planetary health: A systematic review. Int. J. Environ. Res. Public Health.

[46] Murray D.R.P. (1968). The importance of water in the normal growth of larvae of *Tenebrio molitor*. Entomol. Exp. Et Appl..

[47] Zachariassen K. (1996). The water conserving physiological compromise of desert insects. Eur. J. Entomol..

[48] Steinfeld H., Gerber P., Wassenaar T.D., Castel V., Rosales M., de Haan C. (2006). Livestock’s Long Shadow: Environmental Issues and Options.

[49] Piffer P.R., Tambosi L.R., Uriarte M. (2022). Achieving sustainable water and land use systems in highly developed tropical landscapes. Environ. Res. Lett..

[50] FAO (2013). Edible Insects. Future Prospects for Food and Feed Security.

[51] Nakagaki B.J., Defoliart G.R. (1991). Comparison of diets for mass-rearing *Acheta domesticus* (Orthoptera: Gryllidae) as a novelty food, and comparison of food conversion efficiency with values reported for livestock. J. Econ. Entomol..

[52] Oonincx D.G.A.B., van Broekhoven S., van Huis A., van Loon J.J.A. (2015). Feed conversion, survival and development, and composition of four insect species on diets composed of food by-products. PLoS ONE.

[53] Halloran A., Roos N., Eilenberg J., Cerutti A., Bruun S. (2016). Life cycle assessment of edible insects for food protein: A review. Agron. Sustain. Dev..

[54] Dobermann D., Swift J.A., Field L.M. (2017). Opportunities and hurdles of edible insects for food and feed. Nutr. Bull..

[55] van Huis A., Oonincx D.G.A.B. (2017). The environmental sustainability of insects as food and feed. A review. Agron. Sustain. Dev..

[56] Ramos-Elorduy J., Moreno J.M.P., Prado E.E., Perez M.A., Otero J.L., de Guevara O.L. (1997). Nutritional value of edible insects from the state of Oaxaca, Mexico. J. Food Compos. Anal..

[57] Vesterlund M., Borisová S., Emilsson E. (2024). Data center excess heat for mealworm farming, an applied analysis for sustainable protein production. Appl. Energy.

[58] Meyer-Rochow V.B., Gahukar R.T., Ghosh S., Jung C. (2021). Chemical composition, nutrient quality and acceptability of edible insects are affected by species, developmental stage, gender, diet, and processing method. Foods.

[59] Roffeis M., Almeida J., Wakefield M.E., Valada T.R., Devic E., Koné N.G., Kenis M., Nacambo S., Fitches E.C., Koko G.K.D. (2017). Life cycle inventory analysis of prospective insect based feed production in West Africa. Sustainability.

[60] van Broekhoven S., Oonincx D.G.A.B., van Huis A., van Loon J.J.A. (2015). Growth performance and feed conversion efficiency of three edible mealworm species (Coleoptera: Tenebrionidae) on diets composed of organic by-products. J. Insect Physiol..

[61] Mancini S., Sogari G., Espinosa Diaz S., Menozzi D., Paci G., Moruzzo R. (2022). Exploring the future of edible insects in Europe. Foods.

[62] Gałęcki R., Zielonka Ł., Zasȩpa M., Gołȩbiowska J., Bakuła T. (2021). Potential utilization of edible insects as an alternative source of protein in animal diets in Poland. Front. Sustain. Food Syst..

[63] Rowe A. (2020). Insects farmed for food and feed—Global scale, practices, and policy. arXiv.

[64] Mahavidanage S., Fuciarelli T.M., Li X., Rollo C.D. (2023). The effects of rearing density on growth, survival, and starvation resistance of the house cricket Acheta domesticus. J. Orthoptera Res..

[65] Bertola M., Mutinelli F. (2021). A systematic review on viruses in mass-reared edible insect species. Viruses.

[66] Oonincx D.G.A.B., van Itterbeeck J., Heetkamp M.J.W., van den Brand H., van Loon J.J.A., van Huis A. (2011). An Exploration on Greenhouse Gas and Ammonia Production by Insect Species Suitable for Animal or Human Consumption. PLoS ONE.

[67] Roy P., Nei D., Orikasa T., Xu Q., Okadome H., Nakamura N., Shiina T. (2009). A review of life cycle assessment (LCA) on some food products. J. Food Eng..

[68] Halloran A., Hanboonsong Y., Roos N., Bruun S. (2017). Life cycle assessment of cricket farming in north-eastern Thailand. J. Clean. Prod..

[69] Dreyer M., Hörtenhuber S., Zollitsch W., Jäger H., Schaden L.-M., Gronauer A., Kral I. (2021). Environmental life cycle assessment of yellow mealworm (*Tenebrio molitor*) production for human consumption in Austria—A comparison of mealworm and broiler as protein source. Int. J. Life Cycle Assess..

[70] Smetana S., Schmitt E., Mathys A. (2019). Sustainable use of *Hermetia illucens* insect biomass for feed and food: Attributional and consequential life cycle assessment. Resour. Conserv. Recycl..

[71] Joensuu K., Silvenius F. (2017). Production of mealworms for human consumption in Finland: A preliminary life cycle assessment. J. Insects Food Feed..

[72] Nikkhah A., Van Haute S., Jovanovic V., Jung H., Dewulf J., Cirkovic Velickovic T., Ghnimi S. (2021). Life cycle assessment of edible insects (*Protaetia brevitarsis seulensis* larvae) as a future protein and fat source. Sci. Rep..

[73] Salomone R., Saija G., Mondello G., Giannetto A., Fasulo S., Savastano D. (2017). Environmental impact of food waste bioconversion by insects: Application of Life Cycle Assessment to process using *Hermetia illucens*. J. Clean. Prod..

